# Interplay between polymorphisms in immune‐regulating genes *GDF15* and *MIR146A* may confer protection against Alzheimer’s disease

**DOI:** 10.1002/alz70861_108861

**Published:** 2025-12-23

**Authors:** Anatoliy I Yashin, Deqing Wu, Konstantin Arbeev, Olivia Bagley, Hongzhe Duan, Rachel Holmes, Svetlana Ukraintseva

**Affiliations:** ^1^ Social Science Research Institute, Duke University, Durham, NC USA; ^2^ Duke University, Durham, NC USA

## Abstract

**Background:**

Infections and neuroinflammation are increasingly recognized as key contributors to the pathogenesis of Alzheimer’s disease (AD). Emerging evidence suggests that the immune‐regulating genes GDF15 and MIR146A are promising targets for developing treatments aimed at reducing the risks of age‐related diseases associated with chronic inflammation, including AD. The shared involvement of these genes in inflammation and immune modulation suggests potential overlap or interaction between respective regulatory networks. Here we hypothesize that interactions between polymorphisms in the *GDF15* and *MIR146A* genes may confer protection against AD.

**Method:**

We analyzed the Framingham Heart Study (FHS) data using logistic regression model with an interaction term in REGENIE software package. AD outcome was defined as a binary variable: group 1 (cases) included participants who survived past age 85 without AD diagnosis (*N* =1,421); group 0 (controls) included participants diagnosed with AD at any age (*N* =257). To account for linkage disequilibrium (LD) and optimize multiple testing correction, we used the PLINK2 clumping procedure, which results in a higher threshold for Bonferroni correction (BCT).

**Result:**

The interaction between SNPs rs1059369 (*GDF15*) and rs2910163 (upstream *MIR146A*) was associated with significantly reduced odds of AD (β = 0.57, *p*‐value = 1.16E‐02, BCT = 2.5E‐02). Similar interaction effect was seen for rs4808795 (*GDF15*) and rs2910163 (*MIR146A*) (β = 0.57, *p* = 1.15E‐02, BCT = 2.5E‐02). The rs1059369 (missense variant) is in strong LD with the intergenic rs4808795 (D’=1, R^2^=0.99), suggesting that the latter may represent the effects of rs1059369 rather than having its own functional significance. SNP rs2910163 (upstream *MIR146A*) and rs2910164 (MIR146A) are also in strong LD: (D’=1, R^2^=0.77). The associations of SNPs rs1059369 and rs2910164 have also been linked to immune and aging‐related traits in separate analyses and can be found in respective publications.

**Conclusion:**

The results of this study support the importance of interactions that microRNAs and *GDF15* may play in AD risk. They also suggest that detected associations of interactions of SNP rs2910163 related to *MIR146A* with rs1059369 and rs4808795 SNPs related to the *GDF15* gene may be considered when developing interventions aiming to protect against AD.

